# Views about Decay of the Teeth—Old and New

**Published:** 1883-05

**Authors:** 


ARTICLE IV.

### VIEWS ABOUT DECAY OF THE TEETH—OLD AND NEW.

Hippocrates, about 400 B. C., drew the attention of doctors to some diseases of chest, neck and ears, the proximate causes of which he thought to be in bad teeth, and which could only be cured by extraction. Also, the seasons seemed to him to have influence over the teeth. He tries to destroy the humors stagnating and accumulated in these organs by means of certain masticatory drugs.

Galenus, about 100 B. C., informs us, first, that the teeth have soft nerves, because they are naked bones and in union with the tongue, and the other soft portions of the oral cavity contribute to the sense of taste. From him we learn for the first time the doctrine of continual nutrition of the teeth which takes place in proportion to the wear. Two abnormal conditions may result from that fact, namely, deficiency and excess of nutrition. The former makes the teeth weaker, brittle and tender. Excess of nutrition causes a kind of inflammation similar to that in the soft tissues. No remedy exists for the first difficulty, as the deficiency in nutrition not only causes teeth to shrink, but also enlarges the pulp cavity; it is a disease of aged persons. The excess of nutrition is found more frequently in younger persons. The deficiency of nutrition one meets in some measure by astringent drugs; the excess of nutrition and the acid, stomachic juices, by bitter tonic remedies. Discolored teeth ought to be treated with drying substances.

A long night of almost absolute intellectual darkness follows during the reign of a fanatical priesthood.

By order of the German Emporer, Louis IV., in 1315, Mondini performed the first public dissection of a corpse, which had to be repeated every five years.

Mondini, Professor of Bologna, is the restorer of anatomy; he wrote an anatomical hand-book, which for two centuries had a classical authority.

Some noteworthy investigations were made by Paul of Aegina, 636 A. D., who in time of general barbarism lived alternately in Rome and Egypt. He advises, for the preservation of the teeth, to guard against deterioration of the food from indigestion, because that causes frequent vomiting, which is very hurtful to the teeth. Therefore, he forbids the use of dry figs, very cold food, etc.

Ebu Sina or Avicenna, about 1000 A. D., was an adherent of the vital theory. From violently pulsating pains he diagnosticates excessive moisture at the root of the tooth. He drills into the tooth so as to empty it, and to bring the remedy immediately to the diseased spot. Narcotic remedies often ruin the teeth. Against the worms, supposed to live in earious teeth, he advises the seeds of stramonium, garlic and onion.

Alex. Beneditti, Professor at Padna, 1506, in enumerating the causes of caries, thinks he has to accuse the use of milk. He is the first writer who mentions the effect of quicksilver on the teeth, from internal and external use.

Capivacci, 1617, cautioned against sudden changes, such as the use of warm and cold food, as nature cannot bear such sudden changes. In mercurial treatment of syphilis, the patient, as soon as an effect in the oral cavity is shown, should carry a piece of gold in the mouth, because the quicksilver adheres to the gold with its particular sympathy.

Claudius Deidatus, about 1600, surgeon of the Bishop of Basel, asked Hildiani, physician of Bern in 1634, what treatment he ought to employ for a nun who had the rheumatism in the right side of the head, and consequently had such violent pains that she even tried to ease them with nitric acid. Thereby she ruined all her teeth, and even the jaw bone commenced to die. A suddenly cured rash of the head seemed the first cause of the trouble, which became changed into melancholy, with digestive troubles; therefore (?) bad humors were probably the proximate cause of the fluxion.

Hildani himself, in order to kill the nerves in carious teeth, recommends nitric or sulphuric acids, but mentions that, if repeated too often, they ruin the teeth.

Musitanus, Professor of Naples, in 1714, says that the worms in teeth grow from their peculiar eggs which the flies and other insects deposit on the food, and these, remaining in the cavities, are hatched by the warmth of the mouth.

In 1728, Peter Fanchard, the famous Paris dentist, the restorer of the dental art, as the French call him, writes in his works about many things which may interest the dentists. He puts himself to much trouble to prove that the worms were the cause of toothache in carious teeth and in salivary calculus, without ever having found them. He supposed all diseases of the teeth to have an internal and external cause. They first attack the external as well as the internal surface. The caries produced by external agents acts on the enamel only.

Krautman, 1738, says: If by any increased supply of acid lymph the teeth are corroded and become carious and soft, there is no other cure except pulling them out. The ferment of acid lymph in the cavity would never lose its strength. Against the supposed worms in dead teeth, he uses the leaves of the "sadetree," boiled with wine, which "smiteth" them together with the bad humors. Acids and cold food are most hurtful to the teeth.

Pfaff, 1755, says the teeth will suffer. To explain the fact why the upper teeth become destroyed sooner than the lower, he asks: Might not the proximity of the upper jaw to the acrid humors in the nose be the cause?

Ovelgrun, in 1771, claimed that strong tea and coffee were the cause of the prevalence of carious teeth, and that the supposed tooth worms were nothing but the seed of stramonium, used for releiving the pain.

Pasch, in 1767, considered surgar very hurtful to the teeth on account of its preparation with lime, and lime, he says, contains a peculiar corrosive acid that destroys the teeth as well as other bones. (!).

John Hunter, in 1780, said that caries of the teeth does not arise from an external injury by the dissolving action of any liquid, but it is a disease that has its origin in the teeth.

Thomas Bell, 1835, thinks that inflammation is the cause of caries. He explains the fact that caries arises on the outside of the enamel by supposing that, being most remote from the nutrient nerves and vessels, it would be least able to resist. The name caries he considers wrong, and he calls it gangrena. Bell also disputes that the breaking down of artificial teeth is analogus to caries, and that caries is produced by external causes.

Landerer, 1837, says that the teeth are not organized and that caries is nothing but a chemical process of decomposition by the liquids of the mouth, and proves that human teeth attached to artificial dentures sometimes show a kind of caries which is like the ordinary one.

Ficinus, 1847, explains the cause of caries by his new theory of parasites. It is a process of putrefaction which is caused by "germs" in the mouth. He supposed these "denticolæ" to originate from the joining of the so-called Buhlman's fibres, which are nothing else but the fibres of leptothrix buccalis. The process of putrefaction, started by the "denticolæ" (tooth germs,) attacks first Nasmyth's membrane, which becomes brownish or black; later, he says, it affects the enamel.

Klenks, 1850, thinks that a "phytoparasite"—protococcus dentalis—softens and destroys the dentine and feeds on its chemical elements.

J. Bruch, 1852, says the following are causes: 1st hereditary; 2d, acquired; and 3d, endemical in consequence of bad air, water, clothing and food (! !)

E. Neumann, 1860, indorses the vital theory. The injurious substances act on the dentine as an irritant, and as a result of it, a decomposition of the tissues follows. The fibrils in the canaliculi become inflamed, the lamels become thicker at the expense of the basis-substance, and at last the canaliculi become obliterated.

Bridgeman, 1863, tries to explain caries by electro magneticism.

Magitot, 1867, declares caries as a mainly chemical process. He accuses the acids in the saliva and food of producing it; he also produces artificial caries by allowing acids to act on the teeth for a certain length of time.

Herz, of Berlin, repeated the experiments of Magitot, but he could obtain nothing but a brownish-yellow coloration of the dentine, and because he could not find in the canaliculi any thing deviating from the normal, he inclines to the view that the change in the teeth in ordinary caries is due to a vital process.

Leber and Rottenstein, 1867, by their observation of caries, found that the canaliculi were dilated and filled with leptothric bucculis. They could not detect its presence in hardened dentine and enamel. But, as the resistance of enamel and dentine is reduced by the softening action of acids, the fungi enter into the interior of the dentine and by their growth, chiefly in the dentine, may hasten still more the advancement of the process of softening and destruction than the simple action of acids could do.

Wedl, 1870, believed that the carious process arises from the sour secretion of the gums, which in children, young persons, women—particularly during pregnancy--is secreted in excess; in consequence of this increasing secretion, the carious process in young persons is more acute, while during old age is more chronic. He has never seen the leptothric buccalis in caries, so that he thinks that they have no direct connection with the origin of the caries. In chronic cases the granules of "leptothric" are nothing but finely distributed fat in the canaliculi.

Baume, 1877, explains caries as a chemical process produced by the influence of certain acids and sour liquids of the mouth, the use of sour fruits, wines, beverages, mineral waters, etc.

Our readers are familiar with the latest fierce fight between the acid and word chemistry men (Watt, Tafts,) inflammation-school (Drs. Abbott, Atkinson, Boedeker,) and germ theory-investigators (Dr. Miller, Underwood, Clark, Stockwell.)* Take your choice, gentlemen ! !—D. W.—*Extracts from the German of Dr. McSchlenker..—New England Journal of Dentistry.*

---

ARTICLE V.

### THE ODONTOLOGICAL SOCIETY OF GREAT BRITIAN.

*Those who recognize the presence of germs in decayed teeth as well proved seem to be split up into the germ-inflammation adherents (Dr. Abbott ?) ; acid-germ, Dr. Miller ; germatony, Drs. A. & B. ; while others Dr. Stockwell, etc., consider the germs as most important, and the other changes as more secondary.

